# Ubiquitous Knockdown of *Mettl3* using TRiP.GL01126 Results in Spermatid Mislocalization During Drosophila Spermatogenesis

**DOI:** 10.17912/micropub.biology.000511

**Published:** 2022-01-18

**Authors:** Lauren Rowe, Antonio L Rockwell

**Affiliations:** 1 Susquehanna University

## Abstract

METTL3*, *the enzyme that catalyzes the m^6^A RNA modification in Drosophila is highly conserved and essential in various eukaryotic organisms. *Mettl3 *and its homologs have been linked to biological processes such as gametogenesis. We focused on characterizing the role of METTL3 in Drosophila spermatogenesis. We used the Gal4-UAS system to ubiquitously knockdown *Mettl3* in both somatic cyst cells and germline cells. Using immunostaining and confocal microscopy, we found spermatid bundles mislocalize in testes that contain the morphologically abnormal swollen apical tip. Our result suggests *Mettl3 *knockdown using TRiP.GL01126 results in spermatogenesis aberrations.

**Figure 1. Ubiquitous Mettl3 knockdown using TRiP.GL01126 results in an accumulation of spermatid bundles within swollen apical tip:**
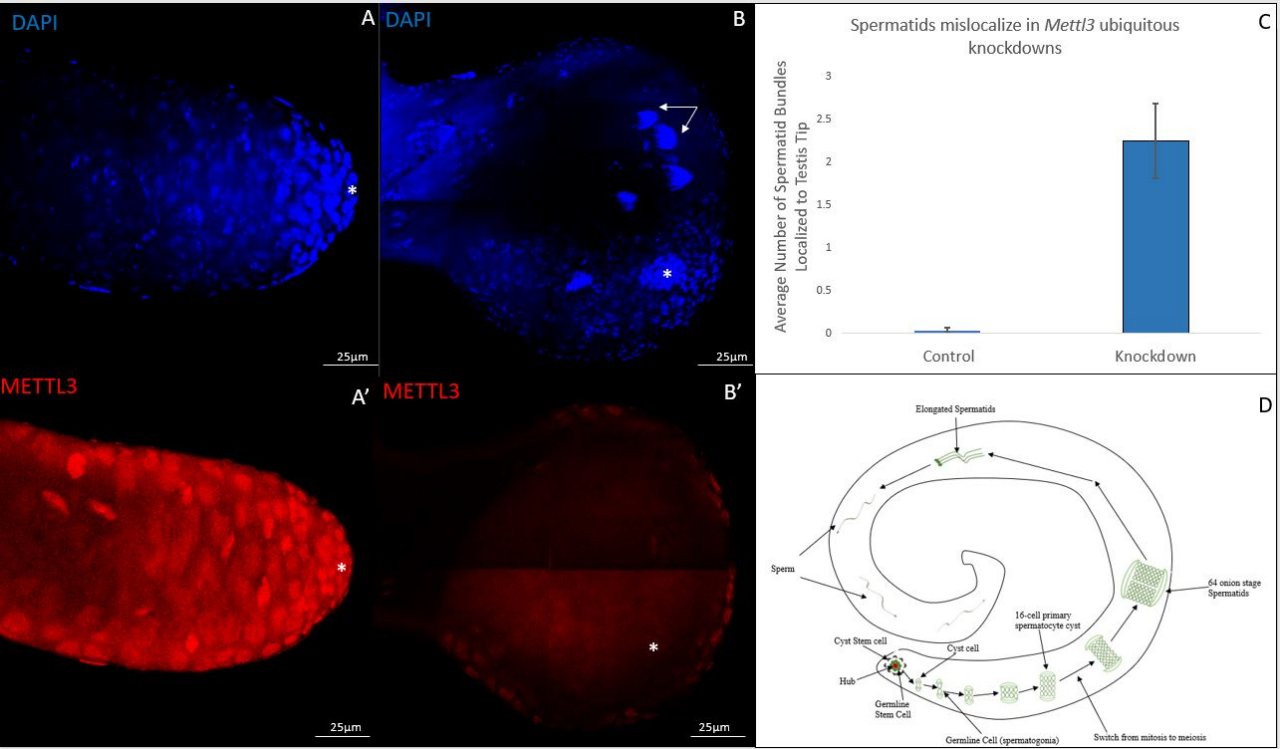
Confocal micrographs. A and B) Control and knockdown testes stained with DAPI. White arrows point at spermatid bundles localized to the tip. A’ and B’) Control and knockdown testes stained with METTL3 antibody. White asterisk indicates hub of testes C) Displays graphic depiction of average number of spermatid bundles between control and ubiquitous knockdown. An ANOVA was performed that revealed a significant difference between the control and knockdown, 0.001>P**. Error bars represent the standard error of the mean (N=30 control, N=22 knockdown). D) Schematic representing the normal stages of Drosophila spermatogenesis. Early stage germline cells are normally localized to the tip while later stage elongated spermatids and sperm are normally localized to the tail of testes.

## Description

METTL3 contains the catalytic subunit of the m^6^A methyltransferase complex. METTL3 serves as a “writer” protein during mRNA processing as it methylates nitrogen at the sixth position of adenosine residues in eukaryotic organisms (Rottman *et al.*, 1994). *Mettl3* and its homologs have been shown to be crucial for developmental events in eukaryotic organisms (Hsu *et al.*, 2017). Developmental processes such as gametogenesis depend on the proper functioning ofMETTL3. Previous research in Drosophila spermatogenesis suggests that somatic cyst cell specific knockdown of *Mettl3* leads to misregulation of an essential protein profilin (chic), which disrupts the somatic permeability barrier (Rockwell and Hongay, 2020). Using spermatogenesis as a model system, our research uses ubiquitous knockdowns to further elucidate the role of METTL3 in spermatogenesis.

Spermatogenesis is an ideal system for characterizing cell localization patterns. In our investigation, we aimed to determine if readily identifiable localization patterns were disrupted in METTL3 deficient testes. To elucidate the role of METTL3 related to our investigation aim, we performed a ubiquitous knockdown to reduce METTL3 in soma and germline cells. We utilized an RU-486 inducible Actin 5C driver system to ubiquitously knockdown *Mettl3*. We dissected testes from balanced controls and ubiquitous knockdowns. We found that 48.8% of knockdown testes had the previously reported swollen tip phenotype (Rockwell and Hongay, 2020). Additionally, we found that the swollen tip phenotype corresponded to an increase in spermatid bundles localizing to the apical tip of the testis (Figure 1A, 1B and 1C) (Wu *et al.*, 2016). In approximately half of the testes with a swollen tip, the hub appeared to move inward from its normal position at the apical tip. We did not see a significant difference in morphologically normal knockdown testes and controls. For all testes, whole-mount antibody staining was done to confirm METTL3 depletion (Figure 1A’ and 1B’).

Germline localization patterns in spermatogenesis have been well characterized. For example, spermatogonia are normally found at the apical tip of the testis while spermatid bundles are normally localized close to tail of the testes (Demarco *et al.*, 2014) (Figure 1D). Our work suggest spermatid bundles in ubiquitous *Mettl3* knockdowns are improperly localizing from their traditional population in the tail to the tip of the testis. The swollen tip phenotype seen in almost half of knockdowns suggests that *Mettl3* potentially plays a critical role in maintaining normal testis morphology as well. Cell mislocalization and tissue expansion phenotypes are associated with numerous developmental conditions. It is important to note that our work involved using a single RNAi construct. Future work using additional RNAi constructs or mutants needs to be conducted to ensure that the phenotypes observed are directly correlated to METTL3 deficiency. If direct correlation is confirmed, additional work will be needed to elucidate the mechanisms responsible for the observed phenotypes in METTL3 deficient backgrounds. Understanding the role of METTL3 will help us gain a greater understanding of the m^6^A epitranscriptomic modification and subsequently biological processes associated with the modification.

## Methods

Drosophila stocks were fed as previously reported (Rockwell and Hongay, 2020).

**Crossing:** *Mettl3 (*FBgn0039139) was ubiquitously knocked down by crossing BL9431 P{ry[+t7.2]=hsFLP}12, y[1] w[*]; P{w[+mC]=UAS-GFP.S65T}Myo31DF[T2]; P{w[+mC]=Act5C(-FRT)GAL4.Switch.PR}3/TM6B, Tb[1] with responder line BL41590 y[1] v[1]; P{y[+t7.7] v[+t1.8]=TRiP.GL01126}attP2/TM3, Sb[1] . Progeny were induced with RU-496 progesterone analog at the L3 stage of development. Inducement prior to L3 resulted in premature death in all progeny containing driver and responder. Progeny were sorted based on tubby and stubble phenotypes.

**Immunostaining:** Young 1-to-3-day oldmale progeny testes were fixed and permeabilized as previously described (Bonaccorsi and Gatti, 2017; Bonaccorsi *et al.*, 2011, 2012). Testes were stained with DAPI as previously described (Rockwell and Hongay, 2020).

**Confocal imagery:** Testes images were obtained from prepared slides using the Nikon-700 series confocal microscope.

## Reagents

**Table:** 

Strain	Genotype	Source
BL9431	P{ry[+t7.2]=hsFLP}12, y[1] w[*]; P{w[+mC]=UAS-GFP.S65T}Myo31DF[T2]; P{w[+mC]=Act5C(-FRT)GAL4.Switch.PR}3/TM6B, Tb[1]	Bloomington Drosophila Stock Center
BL41590	y[1] v[1]; P{y[+t7.7] v[+t1.8]=TRiP.GL01126}attP2/TM3, Sb[1]	Bloomington Drosophila Stock Center
